# Genomic profiling reveals high frequency of DNA repair genetic aberrations in gallbladder cancer

**DOI:** 10.1038/s41598-020-77939-6

**Published:** 2020-12-16

**Authors:** Reham Abdel-Wahab, Timothy A. Yap, Russell Madison, Shubham Pant, Matthew Cooke, Kai Wang, Haitao Zhao, Tanios Bekaii-Saab, Elif Karatas, Lawrence N. Kwong, Funda Meric-Bernstam, Mitesh Borad, Milind Javle

**Affiliations:** 1 Department of Gastrointestinal Medical Oncology, The University of Texas MD Anderson Cancer Center, Houston, TX USA; 2 Investigational Cancer Therapeutics, The University of Texas MD Anderson Cancer Center, Houston, TX USA; 3 Department of Translational Molecular Pathology, The University of Texas MD Anderson Cancer Center, Houston, TX USA; 4 Foundation Medicine, Cambridge, MA USA; 5 OrigiMed, Shanghai, China; 6 Mayo Clinic, Rochester, MN USA; 7 Zhejiang University International Hospital, Hangzhou, China; 8 Peking Union Medical College, Beijing, China; 9 Department of Clinical Oncology, Assiut University Hospital, Faculty of Medicine, Assiut, Egypt; 10 Department of Gastrointestinal Medical Oncology, Unit 426, The University of Texas MD Anderson Cancer Center, 1515 Holcombe Blvd, Houston, TX 77030 USA

**Keywords:** Cancer, Oncology, Genetics, Cancer genomics

## Abstract

DNA repair gene aberrations (GAs) occur in several cancers, may be prognostic and are actionable. We investigated the frequency of DNA repair GAs in gallbladder cancer (GBC), association with tumor mutational burden (TMB), microsatellite instability (MSI), programmed cell death protein 1 (PD-1), and its ligand (PD-L1) expression. Comprehensive genomic profiling (CGP) of 760 GBC was performed. We investigated GAs in 19 DNA repair genes including direct DNA repair genes (*ATM*, *ATR*, *BRCA1*, *BRCA2*, *FANCA*, *FANCD2*, *MLH1*, *MSH2*, *MSH6*, *PALB2*, *POLD1*, *POLE*, *PRKDC*, and *RAD50*) and caretaker genes (*BAP1*, *CDK12*, *MLL3*, *TP53*, and *BLM*) and classified patients into 3 groups based on TMB level: low (< 5.5 mutations/Mb), intermediate (5.5–19.5 mutations/Mb), and high (≥ 19.5 mutations/Mb). We assessed MSI status and PD-1 & PD-L1 expression. 658 (86.6%) had at least 1 actionable GA. Direct DNA repair gene GAs were identified in 109 patients (14.2%), while 476 (62.6%) had GAs in caretaker genes. Both direct and caretaker DNA repair GAs were significantly associated with high TMB (*P* = 0.0005 and 0.0001, respectively). Tumor PD-L1 expression was positive in 119 (15.6%), with 17 (2.2%) being moderate or high. DNA repair GAs are relatively frequent in GBC and associated with coexisting actionable mutations and a high TMB.

## Introduction

Although gallbladder cancer (GBC) is an uncommon malignancy, it represents the most common biliary cancer worldwide^1^. Surgery is potentially curative, however the 5-year overall survival rate is only 60%. Majority of GBC patients are diagnosed with advanced, unresectable disease with 5-year overall survival rates < 5%^2^. Gemcitabine and cisplatin is considered as the accepted first-line systemic chemotherapy regimen for advanced biliary cancers based on the ABC-02 trial. In this study, 36% of enrolled patients had GBC^3^. Next generation sequencing suggests that biliary tract cancers (BTC) has several actionable mutations that include fibroblast growth factor receptor (FGFR), MEK, ERBB2, isocitrate dehydrogenase-1 (IDH1) and DNA repair gene aberrations^4–6^.

DNA repair genes play a critical role in the recognition and repair of DNA-damaging events through base excision repair (BER), nucleotide excision repair (NER), homologous recombination (HR), nonhomologous end-joining (NHEJ), mismatch repair (MMR), Fanconi anemia (FA), and direct reversal (DR) pathways. DNA repair gene aberrations stimulate tumorigenesis, angiogenesis, cellular invasion, and metastasis through genomic instability with subsequent accumulation of several coexisting genetic aberrations, resulting in high tumor mutational burden (TMB)^6,7^. The upregulation of these genes is associated with chemoresistance and radioresistance^8,9^. However, DNA repair alterations have also been associated with a favorable prognosis. Yap et al. determined that patients with muscle-invasive urinary bladder cancer with somatic mutations in 1 or more of DNA repair genes had a higher number of coexisting genetic aberrations (GAs) and a longer recurrence-free survival compared with patients with an intact DNA repair pathway^10^. Similarly, *BRCA2* mutations may have a favorable prognosis in pancreatic cancer^11^.

There are limited data regarding DNA repair alterations in biliary cancers, especially gallbladder cancer. This may be due to the rarity of this cancer, as well as a limited spectrum of next generation sequencing panels used. The Cancer Genome Atlas (TCGA) has listed 193 DNA repair genes, including 122 direct DNA repair genes and 71 caretaker genes that indirectly assist DNA repair through maintenance of genomic stability^12^. Prior studies in biliary cancer focused on only 6 genes: *MSH6*, *BRCA1*, *BRCA2*, *ATM*, *MLH1*, and *MSH*^5,13^. Comprehensive genetic sequencing is likely to further our understanding of this subgroup of patients. There is also a well-described association between DNA repair mutations and immunotherapy reponse, as well as PD-L1 expression^14^. This association has not yet been explored in gallbladder cancer.

## Methods

### Next-generation sequencing

Comprehensive genomic profiling using a next-generation sequencing platform (FoundationOne, Foundation Medicine, Cambridge, MA) at a Clinical Laboratory Improvement Amendments-certified and College of American Pathologists-accredited laboratory (Foundation Medicine) was performed on surgically resected or core biopsy formalin-fixed paraffin-embedded (FFPE) tumor tissue blocks from the primary gallbladder tumor or metastatic lesions from 760 patients with locally advanced or metastatic GBC. All study enrolled patients signed an informed consent form allowing the release of their tissue blocks for molecular testing.

The FFPE tissue blocks were sectioned and stained with hematoxylin and eosin. All slides were reviewed by an expert pathologist to confirm the diagnosis of GBC and that all samples had at least 20% of the DNA derived from tumor cells (Supplemental Figs. 1 and 2). At least 50 ng of DNA per specimen was isolated and sequenced to a high, uniform coverage depth greater than 550 × (mean 759 ×) on an Illumina HiSeq 2000 (San Diego, CA) platform. DNA extracted from the FFPE tumor samples was analyzed by hybridization capture of 3320 exons from 315 cancer-related genes and 37 introns of 14 genes commonly rearranged or altered in cancer. GAs including rearrangements, short variants (base substitutions, short insertions and deletions), and copy number alterations (including focal gene amplifications and homozygous deletions) were determined and reported for each patient sample. The sequencing analysis methods have been previously described in detail^15^.

To classify the identified GAs into actionable/druggable alterations or nonactionable genes, we referenced the actionable genes list prepared by the Institute for Personalized Cancer Therapy-Precision Oncology Decision Support team at The University of Texas MD Anderson Cancer Center^16^. Accordingly, a clinically relevant actionable GA was defined as any GA that can be directly or indirectly targeted with therapies that are approved by the FDA or still under investigation in clinical trials.

Moreover, we assessed the presence of the 19 most common DNA repair GAs previously identified in various cancers^12^. These genes included direct DNA repair genes (*ATM*, *ATR*, *BRCA1*, *BRCA2*, *FANCA*, *FANCD2*, *MLH1*, *MSH2*, *MSH6*, *PALB2*, *POLD1*, *POLE*, *PRKDC*, and *RAD50*) and caretaker genes (*BAP1*, *CDK12*, *MLL3*, *TP53*, and *BLM*).

### Tumor mutational burden

TMB is a marker for genomic instability that is measured by sequencing the whole tumor genome and counting the total number of reported somatic mutations per coding area (megabase) of the examined genome and then dividing it by the size of the tested megabase. We measured the TMB for all tissue samples classified the TMB as low (TMB-L) if the number of mutations per megabase (mut/mb) was less than 5.5, intermediate (TMB-I) if the number of mutations per megabase was between 5.5 and 19.5, or high (TMB-H) if the number of mutations per megabase was 19.5 or higher^17^.

### Microsatellite instability

MSI is another marker for genomic instability. We determined the MSI status in 551 tissue samples by a computational algorithm examining 114 intronic homopolymer loci that were previously selected from 1897 different loci^17^. According to the MSI score, we classified the samples as MSI high, defined as instability in 2 or more microsatellite loci; MSI low, defined as instability in only 1 loci; and microsatellite stable (MSS), defined as absence of any evidence of microsatellite loci instability. If the results for a sample were ambiguous, the analysis was performed a second time^18^.

### Programmed cell death protein 1 and programmed death-ligand 1 immunohistochemistry ventana PD-L1 (SP142) assay

This assay provides a qualitative IHC assessment utilizing a rabbit monoclonal anti-PD-L1 clone Sp142. 83 patients underwent testing utilizing this assay and had tissue stained with OptiView DAB IHC Detection Kit and OptiView Amplification kit on VENTANA BenchMark ULTRA instrument. Patients underwent testing using this assay to determine protein expression by proportion of tumor area occupied by PD-L1 expressing tumor infiltrating immune cells of any intensity or the percentage of PD-L1 expressing tumor cells of any intensity. Raw score percentage of PD-1 positive protein expression was reported per patient as either (a) negative (0% expression), (b) positive—low (1–24%), (c) moderate (25–49%), and (d) high (≥ 50%).

### PD-1 (NAT105) mouse monoclonal antibody

This antibody was utilized to provide a qualitative IHC assessment utilizing a mouse monoclonal anti-PD-1 clone NAT105. 68 patients underwent IHC testing was conducted utilizing VENTANA’s BenchMARK IHC/ISH instrumentation in combination with VENTANA detection kits and accessories. Protein expression was measured in tumor cells and tumor infiltrating immune cells in the specimen. Raw score percentage of PD-1 positive protein expression was reported per patient as either (a) negative (0% expression), (b) positive—low (1–24%), (c) moderate (25–49%), and (d) high (≥ 50%).

### Statistical analysis

Statistical analysis was performed using IBM SPSS version 23.0 (Armonk, NY). *P* values greater than 0.05 were considered significant. Univariate analysis was done using the Fisher exact test for categorical variables and the Kruskal–Wallis test for continuous variables. A box and whisker plot was done to determine the distribution of TMB among all GBC patients. Moreover, we assessed the correlation between DNA repair GAs and TMB and identified the most commonly coexisting actionable mutations in patients with DNA repair GAs. Finally, we described index cases (N = 3) with such alterations and response to matched molecularly targeted therapy.

### Compliance with ethical standards

All procedures performed in studies involving human participants were approved by Institutional research committee (MD Anderson Cancer Center IRB), and were in accordance with the 1964 Helsinki declaration and its later amendments or comparable ethical standards.

## Results

### Comprehensive genomic profiling, tumor mutational burden, microsatellite instability, and PD1& PDL1 expression

Of the GBC patients studied, 525 (69%) were female and 235 (31%) were male. The median age was 64 years (range 25–89 + years). Of the 760 sequenced tissue samples, 371 (48.8%) were from primary gallbladder tumors and the remaining 389 (51.2%) were from metastatic lesions in the liver (21.7%), lymph nodes (6.2%), peritoneum (4.9%), or other distant sites (Supplemental Table 1).

Comprehensive genomic profiling identified 3765 GAs in 760 tumors (Appendix 1). Each tumor harbored at least 1 GA, with an average (mean) of 5 GA per tumor (range 1–46 GAs per tumor). The most frequently altered genes, defined as GAs present in more than 5% of our cohort, were tumor protein 53 (*TP53*; *61.0*%), cyclin-dependent kinase inhibitors 2A (*CDKN2A*; *28.6*%) and 2B (*CDKN2B*; 18.2%), AT-rich interactive domain-containing protein 1A (*ARID1A*; 16.4%), *SMAD4* (15.8%), *ERBB2* (13.9%), *KRAS* (13.2%), phosphatidylinositol 3-kinase CA (*PIK3CA*; 13.4%), mouse double minute 2 homolog (*MDM2*, 11.2%), cyclin E1 (*CCNE1*; 10.9%), *STK11* (9.9%), *ARID2* (8.4%), *ERBB3* (7.8%), fibroblast growth factor receptor substrate 2 (*FRS2*; 9.1%), *MYC* (6.7%), ataxia-telangiectasia mutated (*ATM*; 6.1%), *APC* (5.1%), and phosphatase and tensin homolog (*PTEN*; 5.5%).

**Figure 1:**
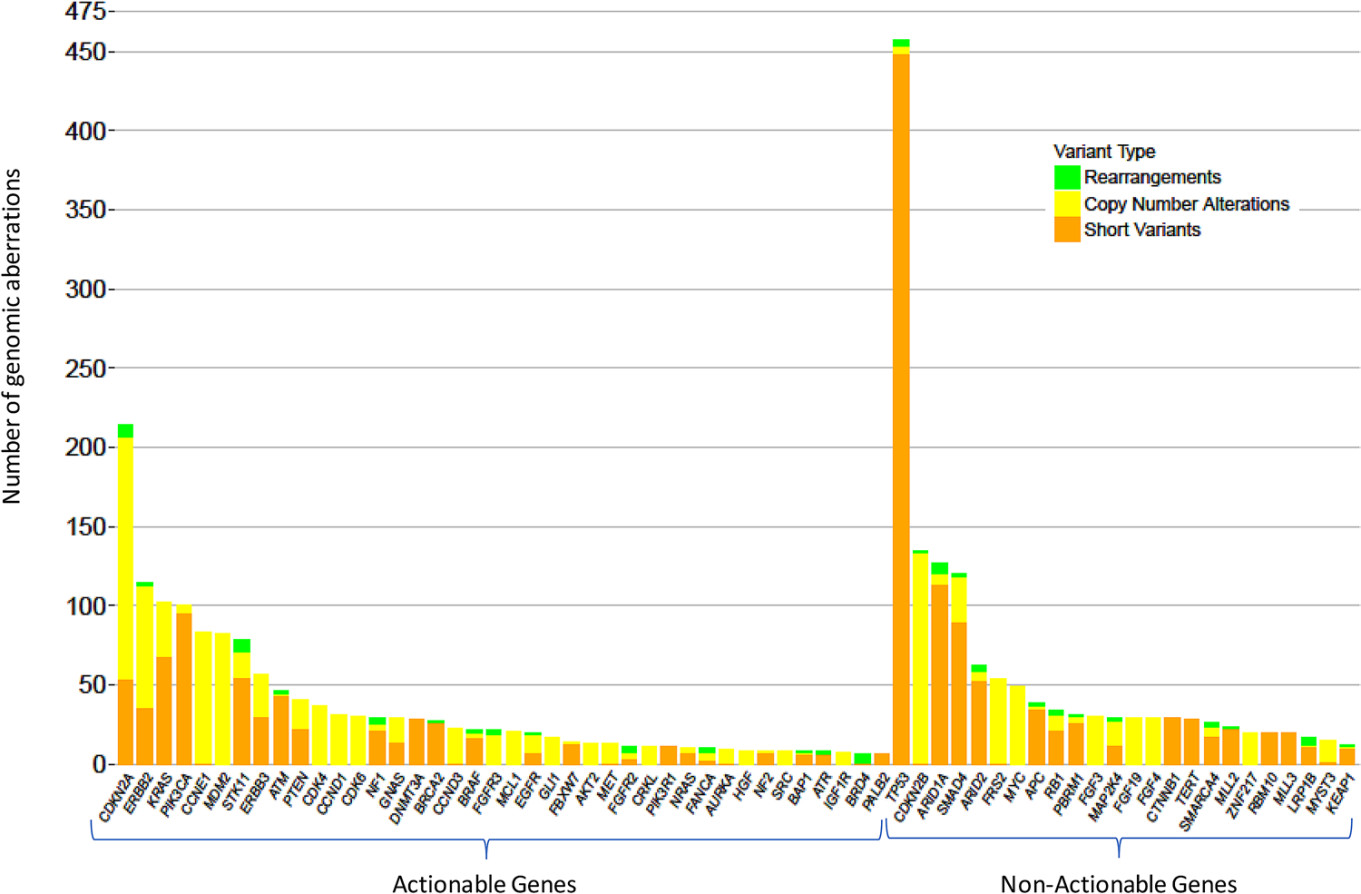
Most common genomic aberrations in 760 patients with gallbladder cancer.

Of the 3765 GAs, we identified 1620 (43%) potentially actionable GAs. At least 1 of these potentially actionable aberrations was identified in 658 patients (86.6%). The most frequent actionable GA was *CDKN2A*, followed by *ERBB2*, *PIK3CA*, *MDM2*, *CCNE1*, *STK11*, *ERBB3*, *ATM*, and *PTEN* (Fig. 1).

**Figure 2:**
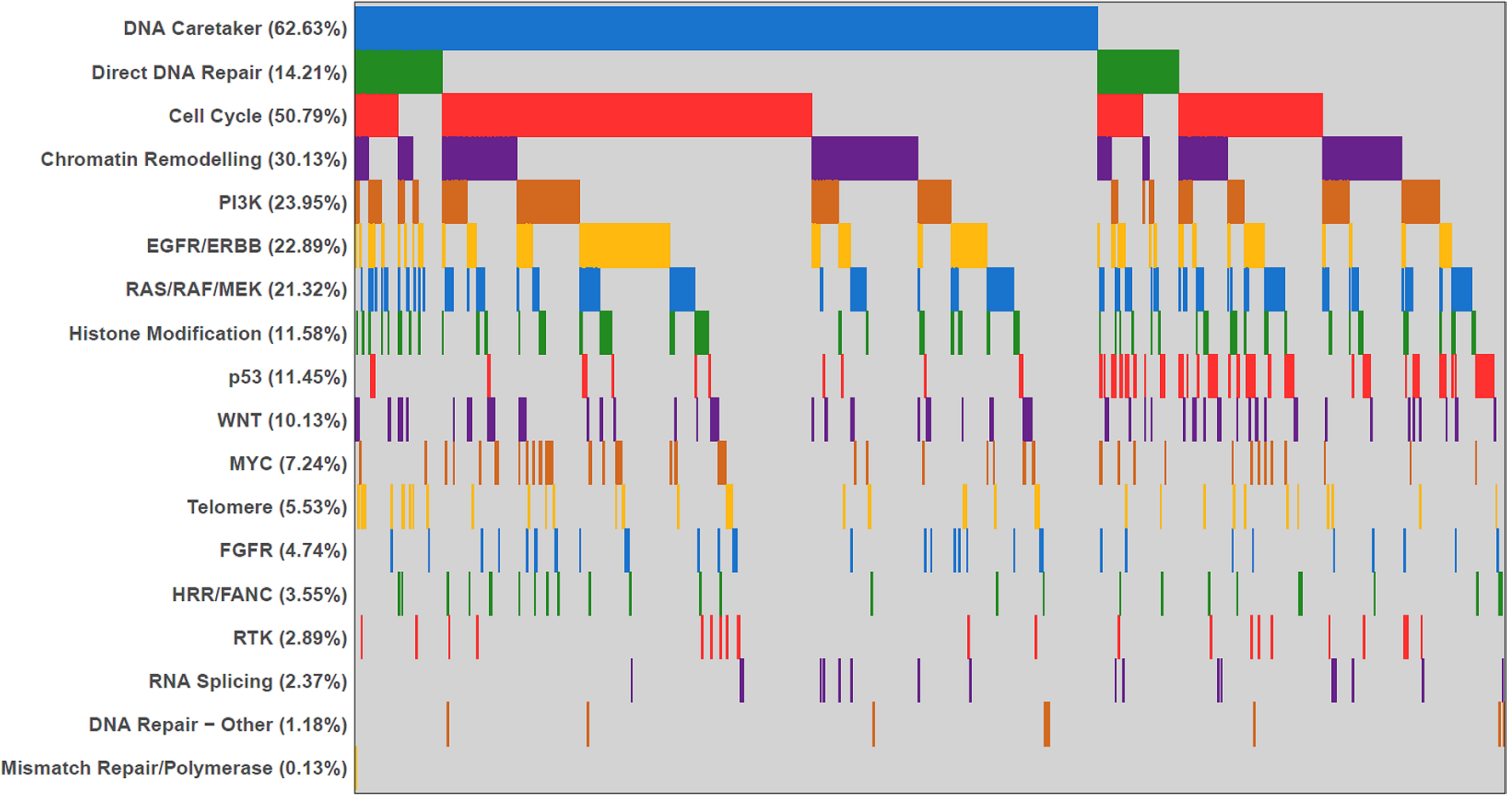
Most common altered genomic pathways in 760 patients with gallbladder cancer.

DNA repair gene GAs were identified in 528 tumors (69.4%) including direct DNA repair gene GAs in 109 tumors (14.2%), predominantly *ATM*. GAs in caretaker genes were identified in 476 tumors (62.6%), predominately in *TP53* (Supplemental Fig. 3). We used lollipop figures to illustrate the distribution of different pathogenic variants of both *ATM* and *BRCA2*, the most commonly mutated direct DNA repair genes (Supplemental Fig. 4). Other commonly altered genomic pathways identified in GBC patients were cell cycle signaling (50.8%), chromatin remodeling (30.1%), PI3K (23.95%), EGFR/ERBB (22.9%), and RAS/RAF/MEK pathway (21.3%) (Fig. 2).

**Figure 3:**
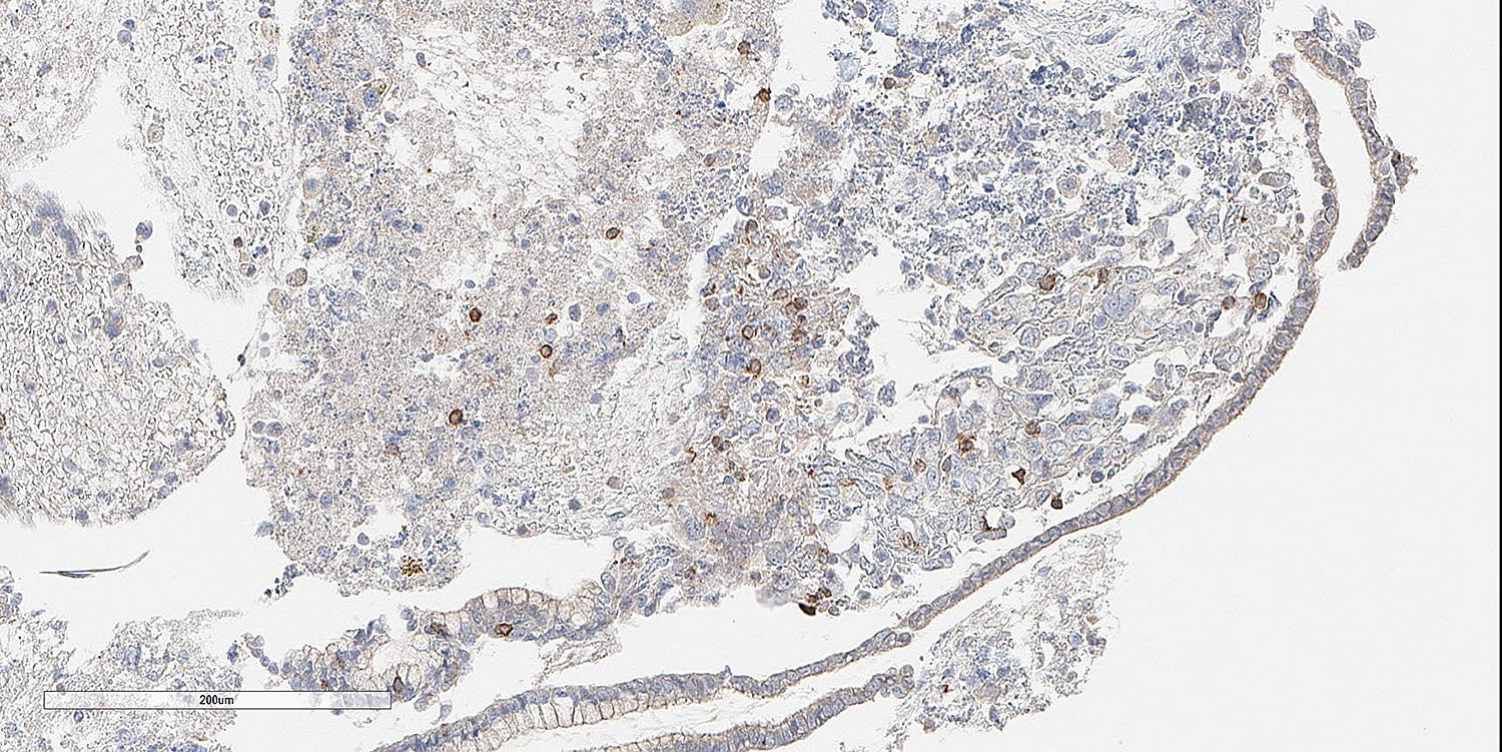
Photomicrograph on an immunohistochemistry stained case of gallbladder adenocarcinoma in a 63-year old man showing low positive staining results for anti-PD-L1 in intratumoral immunocytes. This tumor featured an ERBB2 extracellular domain S310Y mutation. (peroxidase-anti-peroxidase X 200).

**Figure 4:**
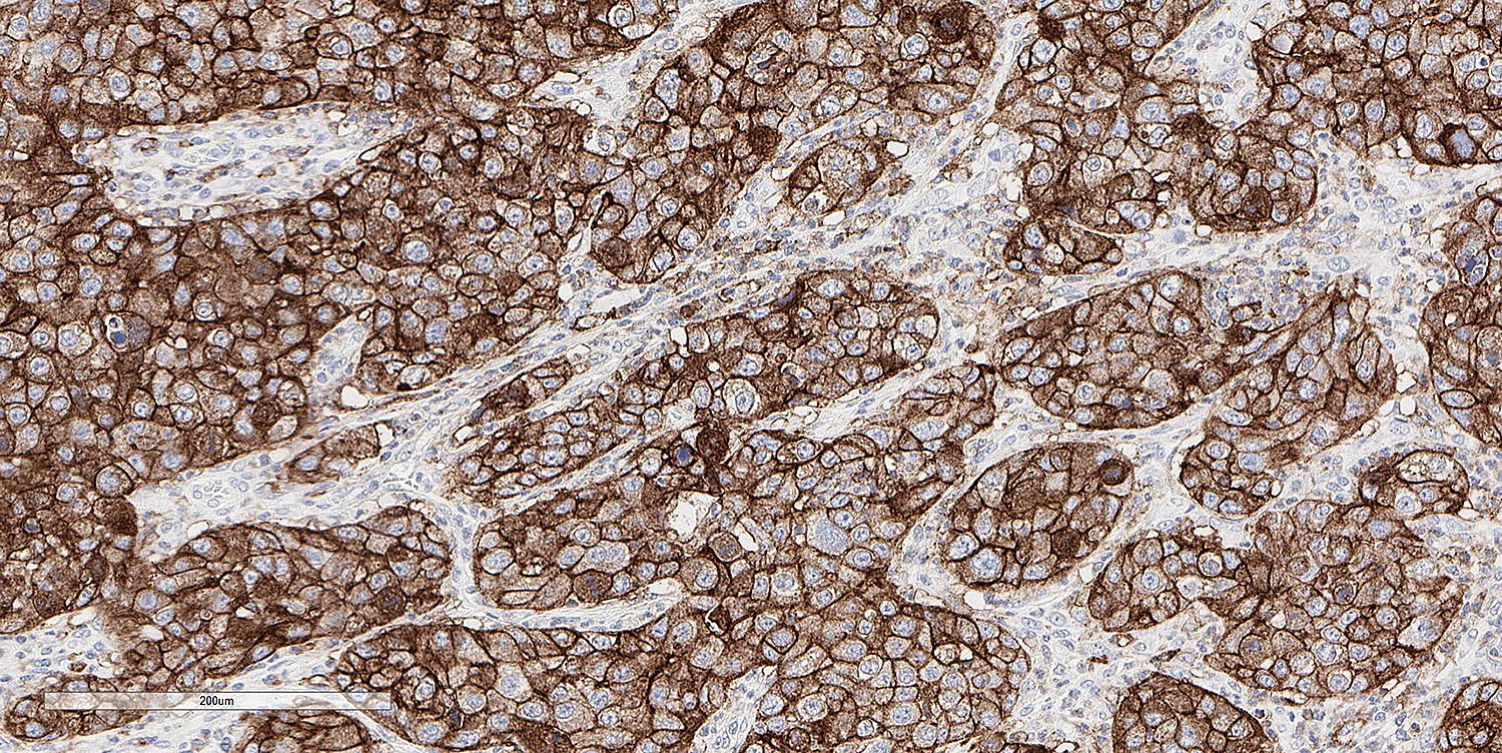
Another gallbladder adenocarcinoma in a 51-year old woman showing 100% high positive staining for PD-L1 in tumor cells. Note the continuous membrane staining pattern. Among other alterations, this tumor featured an STK11 loss. (peroxidase-anti-peroxidaseX200).

The median TMB among the 760 GBC patients was 2.6 mut/mb (range, 0–403 m/mb); 1.2% of patients had TMB-H, 18.4% had TMB-I, and 80.4% had TMB-L (Supplemental Fig. 5). We assessed MSI status in 551 tumors and found that 543 (98.5%) were microsatellite stable, 3 (0.5%) were MSI high, and 5 (0.9%) had unknown or ambiguous MSI status. We evaluated the expression of PD-1 in the tumor-infiltrating lymphocytes (TIL) in 68 patients and PD-L1 in both TIL and the tumor in 82 patients. A total of 83 patients were evaluated and positivity for PD-1, PD-L1 in the tumor, and PD-L1 in the TIL was seen in 51.8%, 15.7%, and 13.3%, respectively (Table 1, Figs. 3 and 4).

**Table 1:** Frequency of PD-1 and PD-L1 in 83 gallbladder cancer patients.

Immunohistochemistry score	PD-1 N = 83 (%)	PD-L1 in the tumor N = 83 (%)	PD-L1 in TIL N = 83 (%)
Negative	25 (30.1%)	69 (83.1%)	71 (85.5%)
Low positive	33 (39.8%)	11 (13.3%)	11 (13.3%)
Moderately positive	5 (6%)	1 (1.2%)	0
Highly positive	5 (6%)	1 (1.2%)	0

*PD-1* Programmed cell death protein 1, *PD-L1* programmed death-ligand 1, *TIL* tumor-infiltrating lymphocytes.

### DNA repair genetic aberrations and tumor mutational burden

GAs in both direct DNA repair genes and caretaker genes were significantly associated with higher TMB. Table 2 shows that patients with either direct DNA repair gene GAs only, caretaker DNA repair genes GAs, or both, had a significantly higher TMB-I and TMB-H as compared with those without DNA repair gene GAs (*P* = 0.004 and 0.002, respectively) (Table 2).

**Table 2:** Associations between DNA repair genetic aberrations and tumor mutational burden.

TMB status	Total	Direct Only	Caretaker Only	Both	Neither	P-value
	N = 760 (%)	N = 52(%)	N = 419(%)	N = 57(%)	N = 232(%)	
TMB-L	611 (80.4%)	44 (84.6%)	326 (77.8%)	35 (61.4%)	206 (88.8%)	0.2
TMB-I	140 (18.4%)	7 (13.5%)	89 (21.2%)	18 (31.6%)	26 (11.2%)	0.004
TMB-H	9 (1.2%)	1 (1.9%)	4 (0.95%)	4 (7.02%)	0.00%	0.0002

*TMB* tumor mutational burden, *TMB-H* high TMB (≥ 19.5 mut/mb), *TMB-I* intermediate TMB (5.5–19.5 mut/mb), *TMB-L* low TMB (< 5.5 mut/mb).

### DNA repair genetic aberrations and coexisting targetable alterations

**Figure 5:**
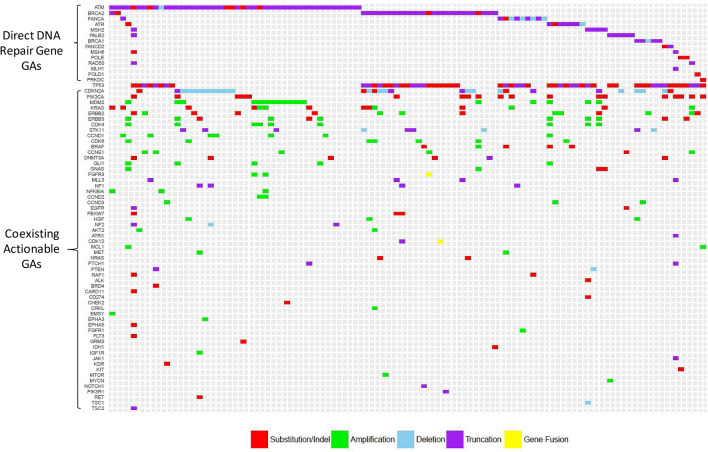
Tile plot for 109 gallbladder cancer patients with direct DNA repair genetic aberrations and coexisting actionable genetic aberrations.

Among 109 tumors of GBC with direct DNA repair GAs, the most common coexisting actionable GAs were *CDKN2A* (n = 31; 28.4%), *PIK3CA* (n = 19; 17.4%), *MDM2* (n = 18; 16.5%), *KRAS* (n = 14; 12.8%), *ERRB2* (n = 14; 12.8%), *ERBB3* (n = 11; 10.1%), *STK11* (n = 8; 7.3%), *CDK4* (n = 9; 8.3%), *CCND1* (n = 7; 6.4%), *CDK6* (n = 7; 6.4%), *DNMT3A* (n = 6; 5.5%), and *CCNE1* (n = 6; 5.5%) (Fig. 5). In 476 tumors with caretaker GAs, the most common actionable GAs were *CDKN2A* (n = 134; 28.2%), *ERBB2* (n = 87; 18.3%), *CCNE1* (n = 77; 16.2%), *PIK3CA* (n = 55; 11.6%), *KRAS* (n = 52; 10.9%), *STK11* (n = 38; 8%), *ERBB3* (n = 31; 6.5%), *PTEN* (n = 30,;6.3%), *CDK6* (n = 25; 5.3%), and *CCND1* (n = 24; 5.04%). In 232 tumors without direct or caretaker DNA repair GAs, the most commonly actionable mutations were *CDKN2A/B, MDM2, ARID1A, KRAS, PIK3CA, and SMAD4.* (Supplemental Fig. 6).

While detailed medical history of subjects included in this study were unavailable, we are including three cases (Supplemental Figs. 7–9): two that illustrate the natural history of DNA repair GAs and one that illustrates outcome with targeted therapy.

## Discussion

Gallbladder cancer is regarded as an ‘orphan’ cancer that is often diagnosed at an advanced disease stage and has very few therapeutic options beyond systemic therapy. This disease occurs in geographic pockets that have limited resources, further limiting clinical and translational research. Identification of novel targets that have therapeutic options has the potential to incentivize clinical research investment in this disease. In our study, we investigated the comprehensive genomic profile of 760 GBC cases to identify the most common GAs, the frequency of the DNA repair genes, and the association between DNA repair GAs, TMB, MSI, and coexisting somatic mutations. Genomic instability is the hallmark for cancer development and progression and further investigation of the same in BTC is critical. Chae et al., noted that the 19 most common mutated DNA repair genes in breast, lung, liver, intestinal, and skin cancers were the direct DNA repair genes *ATM, ATR, BRCA1, BRCA2, FANCA, FANCD2, MLH1, MSH2, MSH6, PALB2, POLD1, POLE, PRKDC, and RAD50* and the caretaker genes *BAP1, CDK12, MLL3, TP53,* and *BLM*^12^. Mutations in all these genes were identified in our cohort.

Direct DNA repair genes regulate the previously described repair pathways. While the BER pathway is responsible for repairing DNA single-strand breaks, both the HR and NHEJ pathways are responsible for repairing DNA double-strand breaks. MMR genes (*MLH1, MLH3, MSH2, MSH3, MSH6, PMS1,* and *PMS2*) are essential for repairing inappropriate nucleotide insertions or deletions as well as base misincorporations. The NER pathway repairs any major helix-distorting damage related to ultraviolet radiation while the FA pathway maintains genomic stability through recognizing and removing DNA interstrand crosslinks, and the DR pathway is essential to eradicating damaging DNA methylation. Dysregulation of these pathways will lead to genomic instability, often with accumulation of several GAs and a higher TMB^9,19,20^.

Our study has noted that a significant proportion of GBC cases (15%) have direct DNA repair GAs, raising intriguing possibilities for DNA repair inhibitors for a fraction of GBC cases. In our cohort, GAs in both direct DNA repair genes and caretaker genes were associated with an intermediate or high TMB. However, only 1.9% and 0.95% of patients with direct DNA repair GAs and caretaker genes GAs, respectively, had high TMB-H, possibly limiting immunotherapy to smaller fraction of these cases. In regards to somatic copy number alterations (SCNAs), cases with either direct or caretake DNA repair GAs had an average of 3.53 SCNAs while those lacking a DNA repair mutations had an average of 2.21 SCNAs (*P* < 0.001). The TMB-H cutoff value remains a topic of debate. Although we defined TMB-H as patients who have at least 20 mut/mb, others reported a lower value as being significant^21^. Yang et al., investigated somatic mutation patterns in paraffin-embedded tumors from a cohort of 108 Chinese and 107 US gallbladder cancer patients. Their study included a panel of actionable somatic mutations whereas the present study was focused on DNA repair genetic alterations. Yang et al. reported that direct DNA repair mutations were more common in Asian as compared with Western patients (30% vs. 11%, respectively)^22^. The list of direct DNA repair genes included in the two studies was similar, with the exception that the present study also included *FANCD2, and MLH1* In the current study, 15% of the patients had direct DNA repair mutations. The frequency of the various direct DNA repair mutations is similar between the two studies with the exception of *ATM* (2% in the Yang study vs. 6% in the current study). These differences may be due to the much larger population included in the present study. The frequency of TMB-H was also different between these two trials (17% in the Yang study vs. 1.2% in the present study). However, it must be noted that TMB-H was defined as ≥ 19.5 mut/mb in the present study and as ≥ 10 mut/mb in the Yang study. Despite these exceptions, we believe that the findings from the Yang study were generally confirmed in the larger cohort represented by the present study.

Several previous studies in melanoma and non-small cell lung cancer showed that high TMB was associated with better response to immunotherapy possibly due to high neoantigen burden^23,24^. Rosenberg et al., assessed TMB for 150 patients with advanced urothelial carcinoma treated with atezolizumab, a humanized monoclonal antibody that selectively binds to PD-L1^25^. TMB was found to be an independent predictor for response to atezolizumab with a significantly higher median TMB among responders (12.4/Mb) compared with non-responders (6.4/Mb; *P* < 0.0001). This median value would have been classified as intermediate TMB in our study and the impact of immunotherapy for intermediate TMB needs to be prospectively investigated.

Our study has several important limitations. Patients did not undergo germline testing for DNA repair GAs. However, the incidence of germline mutations in DNA mismatch repair genes in BTC is < 3% as described in the literature, thus suggesting that the majority of the DNA repair mutations were somatic in our study. Furthermore, correlation between PD-L1 expression and direct DNA repair GAs cannot be definitively examined in this study as only 83 patients were tested for PD-L1 expression and 16% had PD-L1 expression^26^. In this cohort, 13 cases had direct DNA repair GAs. In KEYNOTE-28, a trial assessing the safety and efficacy of pembrolizumab in advanced BTC, Bang et al., reported PD-L1 expression in 42% of patients^27^. There are very few reports on the effectiveness of DNA repair inhibitors in this setting and patients are still most commonly treated with first-line gemcitabine and cisplatin. There is cross resistance between cisplatin and PARP inhibitors, thus limiting the investigation of these agents to platinum-naïve or intolerant cases.

Several therapeutic approaches targeting DNA repair pathways have been developed in recent years, including: PARP inhibitors, ATM and ATR inhibitors, and checkpoint kinase inhibitors^28,29^. Previous studies have also demonstrated that PARP inhibitors, ATR inhibitors, and CHK1/2 inhibitors are potential targeted therapies for *ATM*-mutated cancer^30,31^. Notably, 6.3% of the patients in our cohort of GBC had *ATM* GAs, as compared with a somewhat lower frequency in lung cancer (4.5%) and breast cancer (2.2%)^12^. Among the 109 GBC cases in our study with direct DNA repair GAs, the most commonly identified coexisting actionable GAs were *CDKN2A, PIK3CA, MDM, KRAS, ERRB2, ERBB3, STK11, CDK4, CDK6, CCND1, CDK6, DNMT3A, CCNE1,* and *PTEN*. The impact of these mutations on therapeutic outcome with DNA repair inhibitors is unknown at this time.

To our knowledge, this is the first and the most comprehensive study thus far evaluating the frequency of DNA repair GAs in GBC patients and will hopefully incentivize clinical trials for this subgroup.

## Supplementary information

Supplementary Appendix 1.

Supplementary Legends.

Supplementary Figure 1.

Supplementary Figure 2.

Supplementary Figure 3.

Supplementary Figure 4.

Supplementary Figure 5

Supplementary Figure 6.

Supplementary Figure 7.

Supplementary Figure 8.

Supplementary Figure 9.

Supplementary Table 1.
