# Supplementary material for: Genomic profiling reveals high frequency of DNA repair genetic aberrations in gallbladder cancer
Source: Sci Rep. 2020 Dec 16;10:22087. doi: 10.1038/s41598-020-77939-6 (PMC7745036; doi:10.1038/s41598-020-77939-6)
Supplement: Supplementary file 2 — Supplementary Legends. [file 41598_2020_77939_MOESM2_ESM.docx]

**Supplementary files:**

**Supplemental Table 1:** Patients’ demographic pathological characteristics.

**Supplemental Figure 1:** High Tumor Nuclear Area (“Purity”) Example. Gallbladder adenocarcinoma in a 75-year old man. High power image of a liver metastasis biopsy showing 90% of the microscopic field nuclear area is derived from malignant tumor cells (hematoxylin and eosin X 200).

**Supplemental Figure 2:** Low Tumor Nuclear Area (“Purity”) Example. Gallbladder adenocarcinoma in a 70-year old woman. High power image of a lung metastasis revealing that the majority of nucleated cells are composed of inflammatory cells an lung parenchymal cells. Only scattered islands of malignant cells are identified. This sample was listed as 20% nuclear area composed of tumor cell nuclei (hematoxylin and eosin X 200).

**Supplemental Figure 3:** Frequency of both direct and indirect DNA repair genetic aberrations in 760 gallbladder cancer patients.

**Supplemental figure 4:** The distribution of the pathogenic variants across *ATM* and *BRCA2* genes. Each lollipop indicates a location where a mutation was detected. If multiple mutations were detected at the same location the lollipop will be longer. Y axis indicates how many mutations were detected at each location. Black lollipops indicate indels. Green lollips are basesub mutations.

**Supplemental figure 5:** Box plot of the tumor mutational burden (TMB) in 760 patients with gallbladder cancer.

**Supplemental figure 6:** Tile plot for 109 gallbladder cancer patients with direct DNA repair genetic aberrations and coexisting actionable genetic aberrations.

**Supplemental figure 7:** A 59-year-old woman was diagnosed with poorly differentiated, T2N1M0 gallbladder adenocarcinoma and underwent surgical resection with no subsequent adjuvant therapy. Nodal recurrence was observed by positron emission tomography-computerized tomography imaging (PET-CT) 6 months later. Genomic profiling of the primary tumor demonstrated *BRCA2, TP53, ERBB2, NF1, FBXW7, CDK12,* and *CREBBP* GAs. Patient refused systemic therapy and remained asymptomatic for > 2 years. Symptomatic disease progression was noted on follow-up CT scans.

(A) Axial PET-CT fusion scan revealed a pathologic fluorodeoxyglucose (FDG) uptake in porta hepatis lymph node.

(B) After 2 years and 9 months without active treatment, axial PET-CT fusion scan revealed progression in the porta hepatis lymph node with no evidence of extrahepatic disease.

**Supplemental figure 8:** A 73-year-old woman presented with a gallbladder mass along with small-bowel obstruction. She underwent partial hepatectomy with hepatoduodenal and aortocaval lymphadenectomy for T2N0M0 poorly differentiated GBC. At that time, no adjuvant therapy recommended. Seven months later, follow-up CT showed an abdominal wall hypervascular metastasis. Surgical resection of the abdominal wall mass with abdominal wall reconstructive surgery was done. Pathological evaluation identified moderately-to-poorly differentiated adenocarcinoma, consistent with primary GBC. Genomic profiling of the metastatic lesion revealed *MLL2, MSH2, TP53, PTEN, RNF43, ARID1A, CHD4, CTCF, GATA2, GATA3, LRP1B, PAX5, PBRM1, and SMAD4* GAs*.* Case was also noted to have TMB-H (30 mut/mb) and MSI-High status. Patient did not receive any systemic therapy and continues to be disease free, 3 years after metastatectomy.

(A) Axial contrast-enhanced CT image shows 1.8 cm midline abdominal wall hypervascular metastasis. (B) Axial contrast-enhanced CT image shows disease free 3 years after metastatectomy.

**Supplemental figure 9:** A 53-year-old man diagnosed with moderately differentiated advanced BTC with liver metastasis. Genomic profiling showed *BRCA1*, *TP53*, *ARID1A*, *CCND3*, and *NOTCH1* GAs, as well as amplification in *FGFR2*, *FGFR3*, *RAF1*, *MYC*, *VEGFA* and loss in *SMAD4* with TMB-I and MSS. Patient received gemcitabine plus cisplatin with stable disease till he developed allergic drug reaction. Thereafter, patient was prescribed olaparib and has received 5 months of therapy thus far with a stable disease.

1. Axial contrast-enhanced CT image demonstrates a low attenuated lesion at the anterior superior hepatic segment 8 extending between the left and right liver lobes measuring 4.9 x 2.6 cm (arrow) before olaparib.
2. (B) After four cycles of olaparib, axial contrast-enhanced CT image demonstrates stability of liver metastasis.
