# Supplementary material for: Genomic profiling reveals high frequency of DNA repair genetic aberrations in gallbladder cancer
Source: Sci Rep. 2020 Dec 16;10:22087. doi: 10.1038/s41598-020-77939-6 (PMC7745036; doi:10.1038/s41598-020-77939-6)
Supplement: Supplementary file 7 — Supplementary Figure 5 [file 41598_2020_77939_MOESM7_ESM.pdf]

Genomic profiling reveals high frequency of DNA repair genetic aberrations in gallbladder cancer.

Authors: Reham Abdel-Wahab, MD, PhD[1,9,10], Timothy Yap, MD, PhD[2] , Russell Madison, PhD[4], Shubham Pant, MD[1,2], Matthew Cooke, PhD[4], Kai Wang, MD, PhD[4,5,7], Zhao Haitao, MD, PhD[8], Tanios Bekaii-Saab, MD[6], Elif Karatas, MD[1], Lawrence Kwong, PhD[3], Funda Meric-Bernstam, MD[2], Mitesh Borad, MD[6], Milind Javle, MD[1*]

Authors Affiliations:

[1]Department of Gastrointestinal Medical Oncology, The University of Texas MD Anderson Cancer Center, Houston, TX, USA

[2]Investigational Cancer Therapeutics, The University of Texas MD Anderson Cancer Center, Houston, TX, USA

[3]Department of Translational Molecular Pathology, The University of Texas MD Anderson Cancer Center, Houston, TX, USA

[4]Foundation Medicine, Cambridge, MA, USA

[5]OrigiMed, Shanghai, China

[6]Mayo Clinic, Rochester, MN, USA

[7]Zhejiang University International Hospital, Hangzhou, China

[8]Peking Union Medical College, Beijing, China

[9]Department of Clinical Oncology, Assiut University Hospital, Assiut, Egypt

[10]Cholangiocarcinoma Foundation, Utah, USA

Correspondence:

Milind Javle, MD, Department of Gastrointestinal Medical Oncology, Unit 426, The University of Texas MD Anderson Cancer Center, 1515 Holcombe Blvd., Houston, TX 77030. Telephone: 713-792-2828; Fax: 713-745-1163; E-mail: mjavle@mdanderson.org

Key Words: gallbladder cancer; DNA repair genetic aberrations; targeted therapy

Word count: 3080

Number of Figures: 5 figures and 9 supplemental figures

Number of Tables: 2 tables and 1 supplemental table

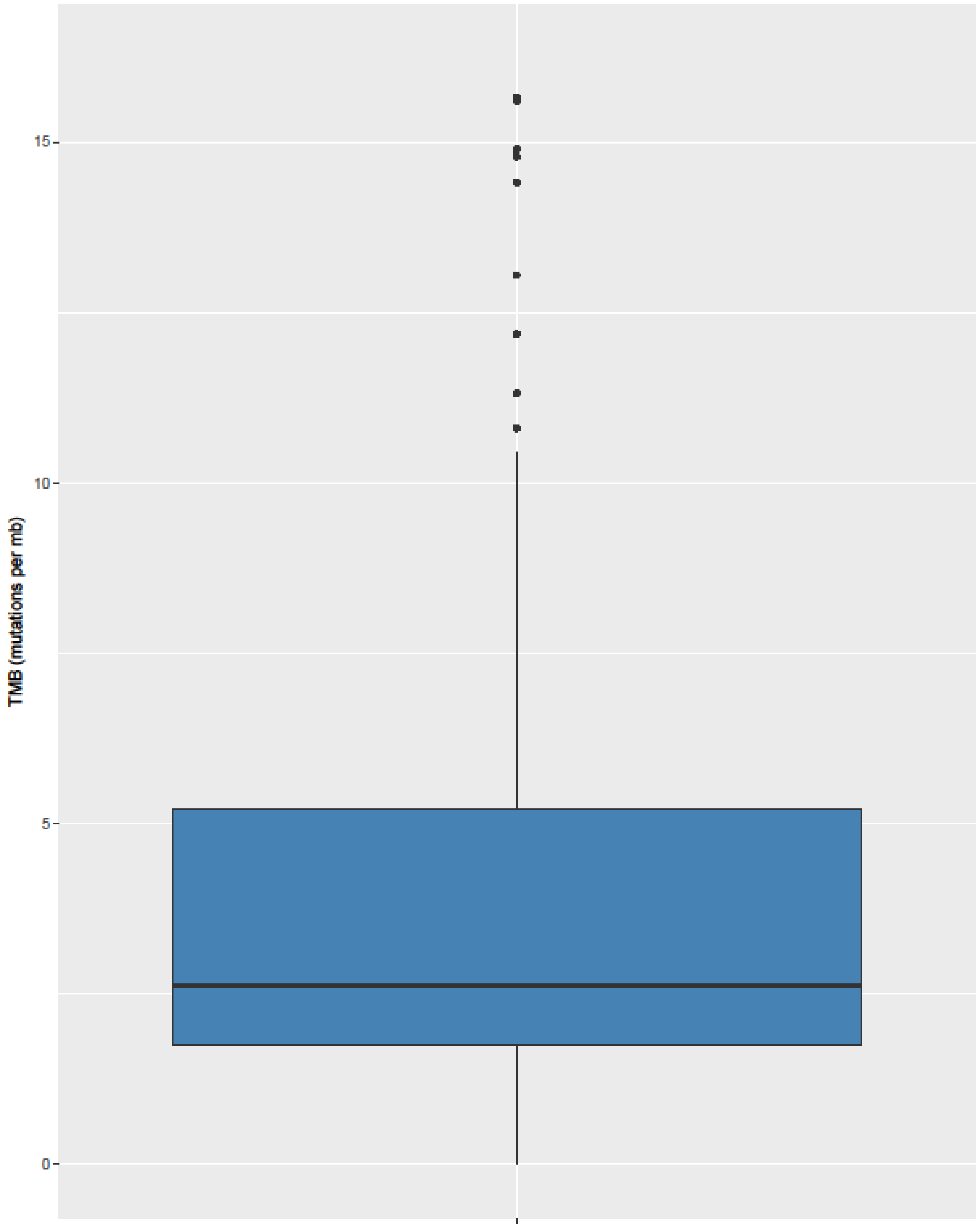
TMB (mutations per mb)
15
10
5
0
