# Supplementary material for: Genomic profiling reveals high frequency of DNA repair genetic aberrations in gallbladder cancer
Source: Sci Rep. 2020 Dec 16;10:22087. doi: 10.1038/s41598-020-77939-6 (PMC7745036; doi:10.1038/s41598-020-77939-6)
Supplement: Supplementary file 8 — Supplementary Figure 6. [file 41598_2020_77939_MOESM8_ESM.pdf]

Genomic profiling reveals high frequency of DNA repair genetic aberrations in gallbladder cancer.

Authors: Reham Abdel-Wahab, MD, PhD[1,9,10], Timothy Yap, MD, PhD[2] , Russell Madison, PhD[4], Shubham Pant, MD[1,2], Matthew Cooke, PhD[4], Kai Wang, MD, PhD[4,5,7], Zhao Haitao, MD, PhD[8], Tanios Bekaii-Saab, MD[6], Elif Karatas, MD[1], Lawrence Kwong, PhD[3], Funda Meric-Bernstam, MD[2], Mitesh Borad, MD[6], Milind Javle, MD[1*]

Authors Affiliations:

[1]Department of Gastrointestinal Medical Oncology, The University of Texas MD Anderson Cancer Center, Houston, TX, USA

[2]Investigational Cancer Therapeutics, The University of Texas MD Anderson Cancer Center, Houston, TX, USA

[3]Department of Translational Molecular Pathology, The University of Texas MD Anderson Cancer Center, Houston, TX, USA

[4]Foundation Medicine, Cambridge, MA, USA

[5]OrigiMed, Shanghai, China

[6]Mayo Clinic, Rochester, MN, USA

[7]Zhejiang University International Hospital, Hangzhou, China

[8]Peking Union Medical College, Beijing, China

[9]Department of Clinical Oncology, Assiut University Hospital, Assiut, Egypt

[10]Cholangiocarcinoma Foundation, Utah, USA

Correspondence:

Milind Javle, MD, Department of Gastrointestinal Medical Oncology, Unit 426, The University of Texas MD Anderson Cancer Center, 1515 Holcombe Blvd., Houston, TX 77030. Telephone: 713-792-2828; Fax: 713-745-1163; E-mail: mjavle@mdanderson.org

Key Words: gallbladder cancer; DNA repair genetic aberrations; targeted therapy

Word count: 3080

Number of Figures: 5 figures and 9 supplemental figures

Number of Tables: 2 tables and 1 supplemental table

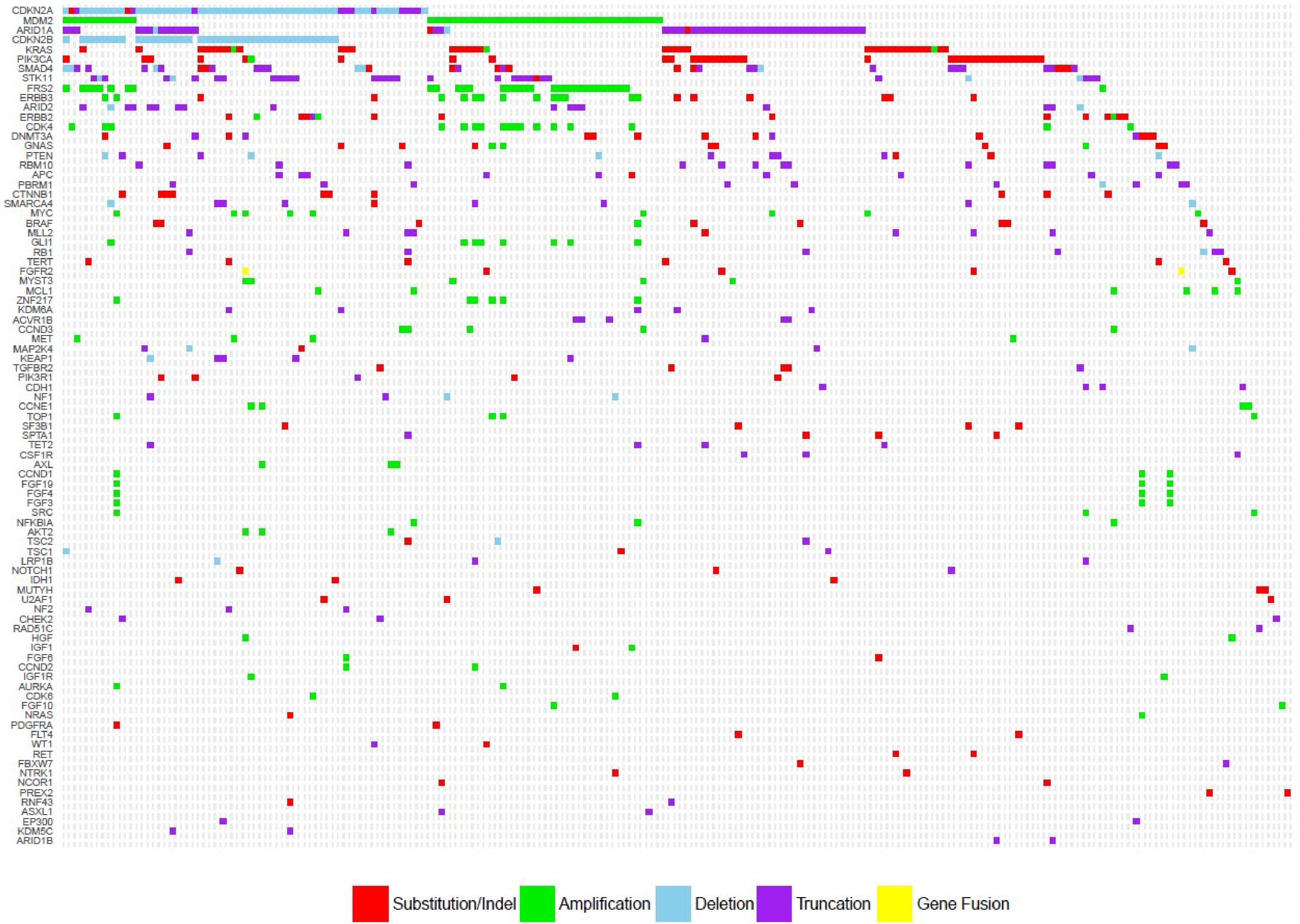
CDKN2A
MDM2
ARID1A
CDKN2B
KRAS
PIK3CA
SMAD4
STK11
FRS2
ERBB3
ARID2
ERBB2
CDK4
DNMT3A
GNAS
PTEN
RBM10
APC
PBRM1
CTNNB1
SMARCA4
MYC
BRAF
MLL2
GLI1
RB1
TERT
FGFR2
MYST3
MCL1
ZNF217
KDM6A
ACVR1B
CCND3
MET
MAP2K4
KEAP1
TGFBR2
PIK3R1
CDH1
NF1
CCNE1
TOP1
SF3B1
SPTA1
TET2
CSF1R
AXL
CCND1
FGF19
FGF4
FGF3
SRC
NFKBIA
AKT2
TSC2
TSC1
LRP1B
NOTCH1
IDH1
MUTYH
U2AF1
NF2
CHEK2
RAD51C
HGF
IGF1
FGF6
CCND2
IGF1R
AURKA
CDK6
FGF10
NRAS
PDGFRA
FLT4
WT1
RET
FBXW7
NTRK1
NCOR1
PREX2
RNF43
ASXL1
EP300
KDM5C
ARID1B
Substitution/Indel
Amplification
Deletion
Truncation
Gene Fusion
