# Supplementary material for: Genomic profiling reveals high frequency of DNA repair genetic aberrations in gallbladder cancer
Source: Sci Rep. 2020 Dec 16;10:22087. doi: 10.1038/s41598-020-77939-6 (PMC7745036; doi:10.1038/s41598-020-77939-6)
Supplement: Supplementary file 12 — Supplementary Table 1. [file 41598_2020_77939_MOESM12_ESM.pdf]

Supplemental Table 1: Patients' demographic pathological characteristics

**Parameter**	**No. (%)**<br>**N = 760**
Age, median (range), y	64 (25-88)
Sex	
Female : male ratio	2.2 : 1
Male	235 (31.0%)
Female	525 (69.0%)
Biopsy site	
Gallbladder	371 (48.8%)
Lymph node	47 (6.2%)
Liver	164 (21.7%)
Soft tissue	21 (2.8%)
Ovary	6 (0.8%)
Peritoneum	37 (4.9%)
Omentum	26 (3.4%)
Bile duct	7 (0.9%)
Pelvis	2 (0.3%)
Appendix	2 (0.3%)
Peritoneal fluid	3 (0.4%)
Iliac crest	1 (0.1%)
Duodenum	5 (0.7%)
Abdomen	6 (0.8%)
Abdominal wall	18 (2.4%)
Muscle	1 (0.1%)
Diaphragm	1 (0.1%)
Pleural fluid	2 (0.3%)
Lung	9 (1.2%)
Skin	2 (0.3%)
Brain	2 (0.3%)
Stomach	1 (0.1%)
Pancreas	2 (0.3%)
Colon	3 (0.4%)
Bone	2 (0.3%)
Fallopian tube	2 (0.3%)
Small Intestine	2 (0.3%)
Adrenal gland	1 (0.1%)
Ampulla of Vater	1 (0.1%)
Retroperitoneum	1 (0.1%)
Unknown	12 (1.6%)
